# Fast Clinical, but Long-Term, Biochemical Remission after Waterhouse–Friderichsen Syndrome

**DOI:** 10.1155/2021/8885348

**Published:** 2021-01-20

**Authors:** Igor Alexander Harsch

**Affiliations:** Department of Internal Medicine II, Division of Endocrinology and Metabolism, Rainweg 68, Saalfeld 07318, Germany

## Abstract

*Background*. The Waterhouse–Friderichsen Syndrome (WFS) is a course of bacterial meningitis with a lethality rate that is still high today. One hallmark of the clinical course is intravascular coagulopathy. This causes hemorrhagic infarctions in the adrenal glands, rapidly causing a primary adrenal insufficiency. Only few reports highlight the course of the remaining adrenal insufficiency or adrenal restitution in survivors. *Case Presentation*. After 3 weeks in an intensive care unit, a 45-year-old male survived WFS with necroses on the legs and forefeet and with primary adrenal insufficiency confirmed by the ACTH stimulation test. The substitution therapy with hydrocortisone and fludrocortisone could be gradually discontinued after nine months due to a further positive clinical course. Although the patient reported good mental and physical performance further on, the cortisol response in ACTH testing showed tiny incremental rises of the stimulated serum cortisol, but to reach a formally normal level, it took about five years. *Discussion*. The report demonstrates a case with a relatively fast clinical remission. A remission of the corticotrophic response occurred in small increments during an observational period of five years. The data suggest that not only a clinical remission is possible but also a complete biochemical remission, although this process may take a much longer timespan.

## 1. Introduction

The Waterhouse–Friderichsen Syndrome (WFS) is a rare course of bacterial meningitis. The most common pathogen is the meningococcus *Neisseria meningitidis*. WFS occurs in about 15% of meningococcal septicemia. The clinical course is characterized not only by the neurological symptoms but also by septic shock with intravascular coagulopathy. Petechial and purpuric lesions of the skin are a classical sign of the disease. Furthermore, the coagulopathy typically causes hemorrhagic infarctions in the adrenal glands, rapidly causing a state of adrenal insufficiency [1]. The disorder typically affects young children [2] or people with functional or anatomical hyposplenia or asplenia [3]. With all options of contemporary intensive care medicine, the lethality is still reported at about 90% [4].

Survivors are often handicapped by the consequences of amputations of the extremities and the primary adrenal insufficiency. Adrenal crisis is reported to occur in about 8% of patients with primary adrenal failure, and such events are unpredictable [5]. We report our experience in a 45-year-old male. A Medline research showed no reports on course and expectable duration of adrenal replacement therapy or the chances for a biochemical remission after WFS.

## 2. Case Presentation

In October 2015, the patient was 45 years old and had been found unconscious but rousable by his colleagues at his workstation. In the emergency room of the nearest clinic, he showed fever, hypotension, and tachycardia, as well as petechiae at the extremities. His mental state soon changed from prostration to coma. He was transferred to the intensive care unit and developed septic shock and multiple organ failure. Vigorous therapy was instituted immediately. He required intubation and mechanical ventilation. Antibiotic therapy was begun with ampicillin/sulbactam; later on, he was treated with ceftriaxone and fosfomycin. High-dose hydrocortisone substitution was initiated, and an Adrenocorticotrophic Hormone (ACTH) test proved pathologic. For circulatory support, catecholamines were necessary for 8 days, and he was in dialysis care for 2 weeks. Meningococci serogroup B could be cultured from blood and cerebrospinal fluid. Necrosis due to the severe circulatory disorder was located at the lateral foot edge, the heels, the toes, and in the interdigital spaces. After one month, the patient could be transferred to a neurological rehabilitation clinic. At the time of transfer, he had a hydrocortisone substitution therapy (30-10-10 mg) and fludrocortisone at 0.1 mg once daily. The patient developed a progressive mummification of the toes. Unfortunately, forefoot amputations had to be performed on both sides.

**Figure 1:**
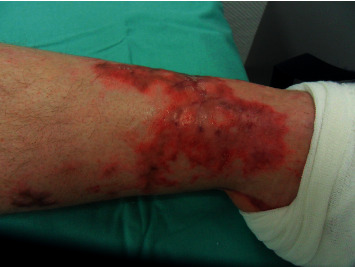
Residuum of tissue necrosis, anterior tibial margin, May 2016.

**Figure 2:**
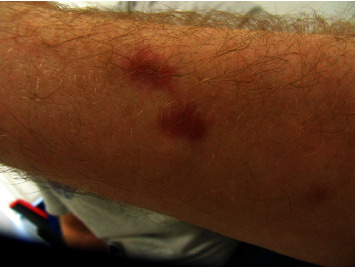
Residuum of skin necrosis, lower thigh, May 2016.

In February 2016, he underwent plastic surgery of the forefeet with grafting with latissimus dorsi flaps to deal with the tissue necrosis. Apart from the functional limitations caused by the tissue necroses (see Figures 1 and 2; 7 months after the onset of the disease), the forefoot amputations, and consecutive reconstructive surgery, his mental and physical performance was good. Under endocrinological supervision, the cortisone replacement therapy was gradually reduced and halted in July 2016. In retrospect, the mode of infection in the patient remained unclear. There was also no evidence of an impairment of the immune system.

ACTH testing in our clinic was first performed in June 2016 with 250 *µ*g corticotropin as recommended by the Endocrine Society [6]. The testing was always begun between 8.30 and 9.00 a.m. We always used 250 *µ*g Tetracosactid (=25 I. E. ACTH) (Synacthen™, Alfasigma S. p. A., Bologna, Italy) for the testing and measured serum cortisol before, as well as 30 and 60 minutes after, ACTH stimulation. Serum cortisol is measured by an Electro-Chemiluminescence Immuno Assay (ECLIA) (Elecsys Cortisol II; COBAS®).

As established by our reference laboratory, a rise of serum cortisol >21 *µ*g/dl is regarded significant. In June, the basal cortisol value was 9.43 *µ*g/dl, 12.4 *µ*g/dl after 30 minutes and 13.8 *µ*g/dl after 60 minutes, respectively.

**Figure 3:**
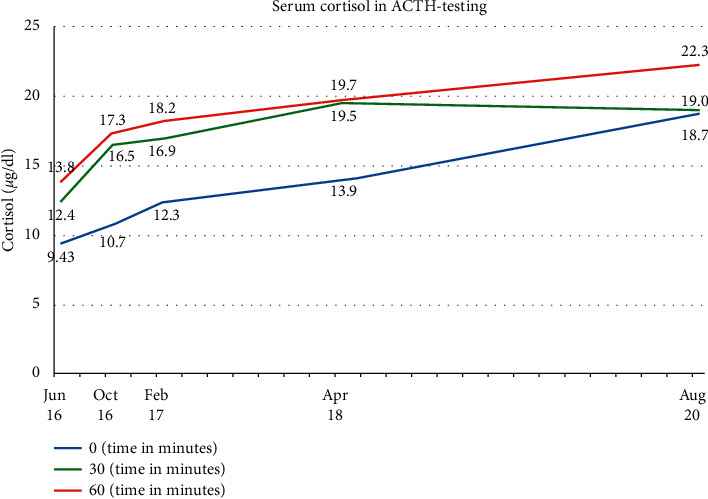
Course of serum cortisol in repetitive ACTH testing from June 2016 to August 2020 (x-axis). The blue lines stand for the basal serum cortisol level, the green line represents the serum levels 30 minutes after ACTH stimulation, and the red line represents the serum levels 60 minutes after ACTH stimulation.

According to the recommendations of the Endocrine Society, peak cortisol levels below 500 nmol/l (∼18 *µ*g/dl) (assay and laboratory dependent) at 30 or 60 minutes indicate adrenal insufficiency. That being said, the patient was initially instructed to keep his emergency card and supply hydrocortisone in case of infection, trauma, and other situations prone to cause adrenal crisis. Such events did not occur in the further course. The patient reported a continuingly good mental and physical performance, and the ACTH test was repeated 4 months after cortisone withdrawal in October 2016 with a basal cortisol value of 10.7 *µ*g/dl, of 16.5 *µ*g/dl after 30 minutes and of 17.3 *µ*g/dl after 60 minutes of ACTH stimulation. The overview of the further ACTH tests carried out shows that there were tiny incremental rises into the range of the normal stimulation values (>21 *µ*g/dl) in a course of five years (Figure 3).

## 3. Discussion

Case reports about WFS focus more on the way the patient's life was saved than on the aftermath. Thus, there is only little published data about the chances of functional adrenocortical remission. The data from our repetitive ACTH-testing over 5 years suggest that a steady regeneration of the zona fasciculata (mainly responsible for cortisol production) after the adrenal trauma caused by WFS is possible.

Slightly different normal ranges for cortisol increases 30 and/or 60 minutes after ACTH stimulation are established between different laboratories. Because of these discrepancies, we do not want to focus the discussion on when exactly a complete biochemical remission of the corticotrophic axis occurred again, but on the recognizable tendency towards an increasingly higher stimulability of the cortisol response, as can be seen in Figure 3. With consistent test conditions (i.e., ACTH from the same manufacturer and using the same measuring methods), a coincidence is largely eliminated. Rather, our observations suggest a very slow but steady reconstitution of the adrenal secretion capacity.

In agreement with this finding and according to the available literature, an “intrinsic plasticity” of adrenal tissue is regarded as established. This assumption is based on the presence of adrenal progenitor stem cells continuously repopulating in the adrenal cortex in a corticotropin-responsive pathway. The first studies on this issue stem from 1938 [7] and 1949 [8] with rat models. The experimental approach, also in more recent studies, is the study of rat adrenal secretion and growth during regeneration after adrenal enucleation [9]. However, this raises the issue of transferability of such results from animal to human.

In our attempt to gain recent data about humans, we found no publications on the issue of adrenal reconstitution after WFS in a Medline research. A largely comparable situation with no further pathogens influencing adrenal function is tuberculous Addison's disease. In a study of 4 cases, a lack of normalization of adrenocortical function after successful antituberculous chemotherapy has been described in a follow-up of 2–5 years [10]. Recently, patients with autoimmune Addison's disease were treated with Tetracosactide (ACTH 1–24) [11]. Only 2 of 11 patients achieved a clinical remission with peak serum concentrations greater than 400 nmol/l where cortisone replacement therapy could be stopped. One of the 2 patients achieved enduring remission; in the other, steroid replacement therapy had to be restarted after week 64. Two other case reports detail the remission of Addison's disease. In the first case of a 39-year-old male, clinical remission was achieved, but even years after withdrawal of hydrocortisone therapy, the rise of cortisol in an ACTH stimulation test was below the normal value [12].

In the second case, a 23-year-old female was on HC for 11 years, and about one year after withdrawal of HC, the stimulated cortisone was 580 nmol/l after 30 minutes [13].

Although the pathophysiology in autoimmune Addison's disease is different from the direct tissue destruction by hemorrhagic infarction or by tuberculosis, the sum of these data favors the hypothesis that the adrenals may be able to regenerate not only to a degree allowing normal mental and physical performance for the patients. This does not necessarily mean that the adrenal secretion is appropriate in situations of stress. However, our data suggest that not only a clinical remission is possible but also a complete biochemical remission, although this process may take a much longer timespan.

## Data Availability

The dataset is within the ORBIS System of our clinic. The laboratory datasets used and analyzed in this case are available from the corresponding author on reasonable request.
